# *Are You Sure This is What You Really Want?* Conversational Recommender Systems for Enhancing Human Autonomy

**DOI:** 10.1007/s11948-026-00619-7

**Published:** 2026-08-08

**Authors:** Francisco Lara, Juan Ignacio del Valle

**Affiliations:** Department of Philosophy I, University of Granada, Granada, Spain

**Keywords:** Recommender systems, Autonomy, Conversational recommendation, Digital ethics, Ethic of recommendation, Socratic assistant

## Abstract

Recommender systems are widely used to help users navigate information overload in digital environments. While they are often portrayed as tools that enhance autonomy by optimizing choice and personalizing content, this article argues that many current recommender systems designs in fact undermine user autonomy. Drawing on a conception of autonomy in which the formation and configuration of preferences, particularly higher-level desires in the Frankfurtian sense, play a prominent role, we examine how dominant recommendation techniques shape users’ desires and, to a greater extent, their identities. We first analyze what we call conjectural recommenders, systems that infer preferences from behavioral data such as clicks or viewing histories. These systems conflate observed choices with genuine preferences, reinforcing first-order desires, narrowing the diversity of recommendations, and trapping users in homogeneous digital environments that impede identity development and degrade deliberative capacities. We then assess interrogative recommenders, which incorporate explicit user feedback as a form of positive friction in the user interface. Although these recommenders prompt users to articulate evaluations, we argue that they remain insufficient for capturing second-order desires and raise new design challenges concerning paternalism and usability. In response, we propose the Socratic conversational recommender system, a model that combines conversational recommendation with a second layer of Socratic questioning aimed at eliciting users’ metapreferences. Rather than prescribing substantive values, the system guides reflection through formal criteria such as coherence, empirical awareness, and cognitive pluralism. The objective is not for the system to discover users’ true preferences but to foster their reflective engagement with what they want to want. We conclude that embedding deliberative dialogue within recommender design offers a promising pathway for aligning algorithmic recommendation with the preservation and development of human autonomy.

## Introduction

Recommender systems (RS) are designed to facilitate users’ decision-making in certain internet services. They are systems composed of algorithms that aim to make suggestions about items that the user might like or find useful. Currently, these applications, after collecting and analyzing data about user preferences and about products and services on the internet, are capable of offering predictions about the possible rating that the user would give to certain items; ultimately indicating to the user the items supposedly best valued by them, in terms of interest or need (Burke et al., 1996; Burke, 1999; Resnick & Varian, 1997).

These RSs are widely used in many internet applications, particularly since the disruption of AI, in its Machine Learning variant, has substantially improved their performance. They are common tools in applications that help users facing information overload in e-commerce, entertainment, education, or social networks. Thus, they suggest items that may interest users, such as certain information, news, music, videos and films, consumer goods, tourist destinations, or people seeking partners. Amazon, Netflix, and Spotify are well-known examples of applications that systematically offer these systems.

At first glance, this use of RSs appears highly relevant to users’ well-being and autonomy. To begin with, given the information overload we encounter when making online choices, these systems are highly effective tools for optimizing choice. Thanks to the internet, we currently have the advantage of being able to acquire information, goods, and services that can be of great interest to us, but that could remain a mere unreachable possibility if we did not have systems that filter that immense quantity of options presented to us and suggest what might interest us most. A human being would hardly have the capacity or time to adequately know all available items and rank them according to their preferences. The RS does this for us, enabling us to acquire what benefits us and what we would have had great difficulty obtaining on our own. But that is not all. By performing the search and selection of what is best for us on our behalf, the system would also allow us to direct the time and effort that this would have required toward other, more gratifying tasks or activities. It is evident that individual freedom is measured not only by a greater range of choices but also by the availability of time. Thus, if the system performs the task of choosing certain items for us, we have more time to use as we wish. Finally, it could also be added that the use of RS would never diminish our human autonomy, since there is always the option of not accepting the system’s recommendations.

This intuitive assessment that RSs, by default, would not only not diminish our autonomy and well-being, but could also increase them, is corroborated by an experimental study concluding that interviewed subjects, internet users of algorithmic decision-making systems, highlight that, in general, with the use of such systems, in addition to never losing control, they increase their choice options and, thanks to the high degree of personalization in recommendations, their desires are better satisfied (Dogruel et al., 2022).

But reality is quite different, because autonomy is more than satisfying one’s desires. It is a complex phenomenon in which the formation and configuration of those very desires have a prominent place. In a basic sense, for desires to be truly autonomous, they should result from a process independent of avoidable external influences; they should be conceivable as belonging to the individual who holds them and should respond, to some degree, to that individual’s rational capacity (Christman, 2020)^1^. It would, therefore, be a questionably simplistic position to maintain that an RS promotes autonomy simply because it satisfies users’ desires. Thus, for example, there would be no advancement in terms of autonomy if the desires that are satisfied are the result of a surreptitious (manipulative) influence to have them, of circumstances that, due to extreme lack of options, condition adaptive preferences, or of a blind and compulsive addiction.

The literature on how RSs actually undermine autonomy is not very extensive, and what exists is highly fragmented. A few years ago, Milano et al. (2020, p. 2), in discussing the ethics of RSs in general, noted the scarcity of literature, citing the novelty of these technologies and the fact that independent researchers cannot access the internal operations of these algorithmic systems due to industrial and commercial secrecy. Currently, although RS technology is no longer so novel and there is greater knowledge of its design, the literature remains scarce, particularly regarding the implications of these systems for human autonomy. This is highly surprising given the importance of autonomy in individual and socio-political terms: it is a constitutive trait of human beings, as well as an indispensable instrument for both personal well-being and development, and for the proper functioning of democratic systems.

Within this scarce existing literature, one part is devoted to studying the extent to which the emergence of RSs can facilitate political and commercial manipulation (Hildebrandt, 2022), especially through the use of hypernudging, that is, the personalization and dynamic reconfiguration of choice architectures based on user feedback and their anticipated preferences (Yeung, 2017). Other issues collateral to the concern about manipulative threats to autonomy are also analyzed, such as the lack of transparency for users regarding the process of obtaining the recommendations they receive (Ricci et al., 2022; Herlocker et al., 2000) or the problems related to privacy during the collection of personal data used by these systems (Friedman et al., 2015; Koene et al., 2015).

These topics, however, fall outside the essential focus of this article. We will instead focus on the risks that RSs pose to autonomy, understood here as the formation of desires in accordance with the basic criteria of authenticity and the exercise of self-control and reflective capacity to act accordingly. Specifically, the objective of this article is twofold. Firstly, to demonstrate that the recommendation techniques used in the main types of RS designed to date endanger autonomy because they bypass and deteriorate the human skills necessary for the deliberative formation of preferences, which in turn constitutes a serious obstacle to developing the user’s personal identity. This, in particular, will be the subject of the following two sections. In the second section, we analyze these undesirable effects in versions of systems that base their recommendations on data about the user’s digital behavior—that is, the user’s activity on social networks or other internet applications. In the third section, we argue that other types of RSs, those that seek to improve their predictions by explicitly querying the user, do not sufficiently address the aforementioned effects on autonomy.

The second main objective of this article is developed in the fourth section, where we attempt to defend that autonomy can only be protected through an uncompromising defense of the “human-in-the-loop” in RS design, and that this is only done adequately through a system-user interaction that makes the user reflect on their preferences. To this end, we will draw on some proposals for so-called positive frictions in the human-machine interface, that is, certain design decisions in these interfaces that precisely seek to break the fluidity in the use of these systems, in our case, to provide time and space for such reflection. A particularly interesting form of friction could be achieved through recent advances in conversational recommender proposals and, above all, through a design that orients these systems toward improving users’ deliberative and self-control capacities. This will be carried out by adapting to the RS domain the Socratic model of moral enhancement proposed in Lara and Deckers (2020) and developed in Lara (2021) for the design of virtual ethical assistants. This model is best understood as one position within a longer-standing debate on AI-assisted moral enhancement. Well before these proposals, Klincewicz (2016) argued that AI could serve moral enhancement through an artificial moral reasoning engine that presents users with arguments grounded in first-order normative theories, while warning that the persuasive force of such a system may rest partly on the undue trust that people place in automated technologies. Klincewicz (2019) subsequently explored how artificial agents might nudge users toward practices of moral self-cultivation, making explicit the trade-off between effective guidance and respect for the user’s autonomy that any AI assistant engaged in dialogue must negotiate, together with the attendant risks of paternalism and manipulation. Alongside related proposals such as the “moral AI” of Savulescu and Maslen (2015) and the “artificial moral advisor” of Giubilini and Savulescu (2018), this literature frames the alternative that Lara and Deckers (2020) later operationalize: between an advisory model, in which the system supplies moral answers, and a Socratic model, in which it merely stimulates the user’s own deliberation. Our proposal takes the Socratic side of this debate; but, as we argue in section ”Socratic RS”, the challenges articulated in that earlier literature, remain pressing for any recommender built along these lines, and we address them directly there.

## Autonomy and Conjectural Recommenders

The main threats to autonomy from the most widespread RSs today arise from the use of a basic approach to designing operations, whereby these systems predict which items the user is supposedly most likely to value. They are designed to record past or current user actions, such as clicks or viewing time on specific items, during their interactions with digital applications. Although several recommendation techniques can be derived from this form of algorithmic design, they can all be encompassed under the title of “implicit feedback RS,” or, as we will call it, “conjectural recommender” (CR).

Underlying this type of recommender is a twofold “conjecture” that makes them appear, in principle, highly efficient: on the one hand, the conjecture that if a certain item has pleased the user because of its content, the user should also like another item with similar content; and on the other hand, the conjecture that the user’s original tastes are evident from the very behavioral facts that support them—that is, from the fact that the user chose them.

However, the main problem with maintaining this scheme of feeding systems primarily with behavioral data is that it ignores the gap between behavior and human preferences. Our choices, including those in the everyday use of digital applications, do not always align with our life plans, essential values, or personal development purposes; in short, choices do not always match what we would want to want. Therefore, if the system continues to recommend similar content to us based on our past or present choices, it will ultimately end up reducing the field of options or opportunities so much (to those remaining on one side of the gap) that it cannot be said to recommend authentic preferences, since it will not count those that do not appear in our choice history (which are on the other side of the gap), and which often better determine what we really want.

In other words, CRs would hinder users’ capacity to form those second-order desires that respond to deeper purposes maintained over extended periods of time, and that, according to H. Frankfurt (1971) and G. Dworkin (1988), would characterize genuine autonomy. The vast majority of RSs are based on a narrow form of human engagement, focused mainly on providing extreme fluidity in their use, exemplified by the one-click buy button, the autocomplete function in search engines, or the automatic video playback. This mainly leads to automation bias: by encouraging immediate reward, this design maximizes uncritical acceptance of proposed recommendations, and hinders the pursuit of preferences through active wanting. This renders us highly passive and distances us from what is distinctly human, characterized by acting through rationalization and reflection on what one does and thereby shaping one’s preferences and determining one’s future (Krook & Blockx, 2023).

Furthermore, by narrowing perspective and repeatedly offering to satisfy immediate desires, the only result is the reinforcement of an addictive tendency in us to always consume the same things. This weakens the user’s self-control capacity and usually leads them to a negative feeling about their own agency, with the consequent remorse for having dedicated so much time to the same thing, normally something very much in line with their most superficial preferences (Burr et al., 2018; de Vries, 2010; Koene et al., 2015). W. Lu (2024) deepens this line of criticism of CRs by arguing that decisions made with personalized algorithms, unlike exclusively human ones, make it difficult for those decisions to be self-corrected. When making decisions in accordance with such algorithms, one would dispense with the traditional correction mechanisms in the non-digital world that help make decisions less biased and therefore more autonomous: social interactions and rational deliberation. That is, because the decisions suggested by the systems are made in a closed environment, users would not be exposed to perspectives different from their own and the system’s—perspectives that could reveal those cognitive biases.

Another of the main criticisms of CRs is directed, above all, at the threats this technique poses to the free development of personal identity. On the one hand, because it causes the user to become “trapped in the past.” The system, by considering the user’s current behavior—for instance, more or less recent clicks or viewings—bases its recommendations on a snapshot of who the user has been or is now, rather than on who the user wants to become in the future. The user’s past desires are strongly reinforced by the CR, even when the user, through second-order desires, had been thinking for some time about changing their desires or, even, had begun at that moment a gradual change that, due to its limited history, does not end up being relevant in the profile the system has assigned to them. It is in this sense that, given the preponderance of past preferences and the insistence on recommendations based on them, it could be said that the user is, in a way, trapped in time (Krook & Blockx, 2023).

On the other hand, this technique is also an impediment to personal development because it traps the user not only in the past but also in the same content. The CR technique renders the user’s digital environment highly homogeneous and immutable, thereby trapping them in a filter bubble that also inhibits personal and intellectual development (Nguyen et al., 2014; Bozdag & Van Den Hoven, 2015).

In short, the blocking of change initiatives in preferences and the consequent absence of new content place the user in an “identity inertia,” whereby the normative principles of the future self tend to be the same as those of the present self (Loi et al., 2020).

Within this category of CRs, we would also include collaborative filtering techniques. In these cases, the aim is to generate recommendations by creating user profiles (profiling) and grouping them into similar clusters, which may be social or demographic categories such as age, gender, marital status, and so on (Yannam et al., 2023).

This could be considered an advance insofar as it would somewhat broaden the range of opportunities and options for the user, since members with common group preferences or categories might also have distinct preferences that would end up being recommended to that user. However, homogenization would persist to a large extent because, even with some novel options, the recommendations would largely reflect the desires of those who are similar to us, but not those of other groups to which we might belong in the future and which would be qualitatively different. Given the similarity among members, the digital environment that will be derived from this type of systems will necessarily be very homogeneous and immutable, and this would reduce the user’s opportunity to discover their potential new interests, preferences, and values, thus limiting their self-knowledge and life experience. This technique, like the previous one, would continue to isolate the user from different lifestyles and deprive them of an update of their preferences. The limited knowledge one has of oneself regarding what one wants and what one can aspire to limits authenticity because it limits the capacity to live according to ideals one would reflectively endorse. Without that broad knowledge of other perspectives with which to compare one’s own, it is impossible to reflectively endorse the values one holds and, ultimately, to be able to claim them as one’s own.

On the other hand, and to make matters worse, the most commonly used collaborative filtering technique is one in which groups are automatically and opaquely created (clustering) and profiles are automatically assigned to them based on a given objective. This filtering technique has proven effective in increasing user engagement; however, by prioritizing only that effectiveness, it introduces another twist to the problems related to the experience and development of personal identity that we have just introduced. The difficulty here lies not only in the fact that the list of characteristics defining these groups (clusters) is opaque, but also in that, even if we could access the internal workings of these algorithms, we would see that the system is not necessarily creating groups related to socially recognizable or meaningful categories (de Vries, 2010). Thus, for example, the system may decide that a group composed of men, fathers, single, aged 43, cat owners, who have watched the film Matrix three times, who are iPhone users but do not own a smartwatch, and a long list of other characteristics, are statistically inclined to consume a certain product. Perhaps a particular user could identify with some of these characteristics if they reflected on their preferences, beliefs, and desires, but they would hardly identify with all of them, and—most importantly—there has been no reflection to select some and not others. This highlights how the use of a recommender system based on collaborative filtering contrasts with how individuals make sense of, and reflexively develop, their own identity through social categories and the reasons and obligations these encompass (Milano et al., 2020; Del Valle & Lara, 2023).

Special mention deserves the criticism that all these types of CR may entail an important degradation of certain capacities of great value for autonomy. For some authors, this is a problem that extends to most uses of virtual assistants, insofar as they involve an outsourcing of important cognitive tasks, which ultimately atrophies the human capacities that enable them (Carr, 2014; Lu, 2024). In the case of CRs, those tasks would be those closely related to deliberative capacities and willpower. This criticism of AI induced deskilling is grounded in long-standing experiments demonstrating that the greater the cognitive effort invested in tasks such as memorization, conceptual understanding, or problem-solving, the greater the efficiency in performing these operations in the future (Slamecka & Graf, 1978). From this follows, as a corollary, degradation as the opposite effect: if the effortful use of certain cognitive tasks improves functions connected to them, their non-use, through delegation to other agents, will also worsen those functions, as certain experiments have demonstrated (van Nimwegen et al., 2006; Burgos et al., 2007; Bastani et al., 2024; Kosmyna et al., 2025).

Some authors have attempted to argue that this is not a problem in itself. That it ultimately depends on the type of tasks that are delegated. To this end, they introduce the distinction between instrumental tasks and those considered intrinsically valuable. The main argument is that if the tasks that are delegated are instrumental (and they acknowledge that the effect on these can indeed be degenerative), we end up with an overal positive final balance, since freeing ourselves from performing certain tedious, repetitive, and unimportant tasks can leave us more time and energy to better (rationally) devote ourselves to intrinsically valuable tasks (Danaher, 2018; Leyer & Schneider, 2021). But to this defense of delegating instrumental tasks, one could object that, regardless of the type of task, cognitive outsourcing could lead to the degradation of highly valuable skills, such as those involved in rational choice (searching, comparing, deliberating, exercising self-control, persevering …). In other words, if we continue to become increasingly dependent on AI assistance, even in more instrumental matters, we run the risk of also losing the capacity to think for ourselves about deeper matters (Carr, 2014; Crawford, 2015; Newport, 2016; Kosmyna et al. 2025). Along similar lines, other authors have raised doubts about the possibility of even making the mere differentiation between instrumental and intrinsically valuable tasks, let alone whether different cognitive capacities can be assigned to one set of problems versus the other (W. Lu, 2024, p. 5).

## Interrogative Recommenders as Positive Frictions and Their Limitations

We have seen the risks to user autonomy posed by recommender systems that operate on the basis of data about users’ activity—data about the immediate desires that the system captures from a history of choices. An important cause of these problems is that RS design prioritizes a smooth user experience that, intentionally or not on the part of developers, results in automation biases, deskilling, or difficulties in reflecting on our second-order desires, as we saw above. This fluidity is one of the main characteristics sought in the design of user-friendly or ergonomic interfaces. In fact, in general, frictions in the human-machine interface—that is, points of difficulty or interruption during user interaction with the system—have traditionally been considered something to avoid in the design of these interfaces. However, recently, certain frictions have begun to be treated as potentially positive “behavioral speed bumps,” especially in situations that require greater self-control, more reflection, and in contexts where automation biases may predominate (Chen & Schmidt, 2024, p. 18). Although they had little impact, the first friction proposals consisted of introducing delays into the various actions a user could take. Other frictions seek to encourage specific actions or alert the user to certain system limitations. Paradigmatic examples of the latter include the “read before you post” on X (formerly Twitter), which counteracts the impulsive publication of hoaxes and misinformation, and the now disappeared warning in ChatGPT below its input box that encouraged users to verify the information provided by the chatbot.

Within the field of RSs, a first positive friction has been to directly ask the user about their evaluation criteria. This is what has been attempted with algorithms based on a design that feeds on data derived from specific evaluations requested from the user. The user must indicate, either numerically or verbally, how much they like a particular item. These recommender systems are known as “explicit feedback” systems, and here we will call them “interrogative recommenders” (IR). This type of RS would represent an advance over those previously discussed because, in addition to resolving the technical problem of the cold start (which occurs when CRs do not have sufficient data on user preferences), they could yield better results regarding autonomy. They would escape some of the criticisms leveled at CRs insofar as, by asking the user to stop and think about their responses to the evaluation request, such responses would be more reflective and aligned with the user’s intentions, thereby reducing the aforementioned gap between these intentions and immediate desires (Angwald et al., 2021).

However, these techniques also do not serve to give the user what they “want to want,” since the user is limited to evaluating only what they already see. If the user only sees romantic movies because the system, based on past history, only recommends items of this film genre, when asked to evaluate them, they will only do so for such items. Still, they will not have the opportunity to tell the system that they “want to want” to watch historical films. For this reason, Krook and Blockx (2023) insist that, in order for the RS to access those second-order desires that do not appear in the user’s history, the user must be given a space and a time within the application to think about whether they really want to choose what they are choosing. That is, they need to be able to question their first-order desires within the context of the system itself.

Other types of IR design aim to advance this objective of allowing some reflection. For instance, IRs based on questions about topics or categories would require greater user effort but would allow them to choose the type of content they want to be shown. Certainly, by confronting the user ex ante with the question of what they want to choose from, we could be making progress toward eliciting in the user a certain second-level preference, since, as in the previous cases, the user would be compelled to stop and think—but in this case, not about what is presented to them, but about other possible content for choosing.

But Krook and Blockx (2023) also see problems with this IR technique. They argue that the system could yield biased results, because the question posed to the user about their preferred content is framed within pre-established categories that may not fully capture all the user’s possible interests. Furthermore, once within those categories, we could not prevent the recommendations for individual items from following biased criteria of popularity or efficiency. Popularity bias manifests when the algorithm disproportionately promotes items with high historical engagement, effectively silencing the “long tail” of diverse and lesser-known content in favor of what is already trending. Simultaneously, efficiency-driven criteria prioritize items that are statistically more likely to yield immediate returns, such as clicks or retention, over those that might offer deeper personal relevance. This biased tendency of the system to recommend certain strategies or items more frequently would then result in greater group conformity and filter bubbles, thereby repeating the drawbacks of excessive homogenization seen in earlier systems.

Increasing the level of friction, designs have also been proposed that promote less unreflective behavior and in which the user acquires more control over the personalization process that gives rise to the recommendation. Regarding the former, it has been proposed to add mechanisms similar to the “delays” mentioned above: an intervention mechanism that forces the user to reflect on and become aware of their excessive use of recommendation systems—for example, by requiring the user to solve certain cognitive tasks if they want to continue using the application, or by allowing them to self-limit the daily time they can access the application (Angwald et al., 2021).

On the other hand, the user can be given greater control over how the system works, by providing them with the possibility or the requirement, at regular intervals, of reconsidering their preferences. Users need to be able to update their preferences whenever they wish, rather than only expressing them when they first enter the application, or even to reset to default values at any time (Krook & Blockx, 2023; Guo, 2018; Knijnenburg et al., 2016). Along similar lines, we have proposed elsewhere that users be provided with the ability to access, in a comprehensible manner, the profile stored by the system and to change characteristics with which they do not identify (del Valle & Lara, 2023). Finally, it has been proposed to include in the system a customizable interface with various degrees of control, allowing users to choose between a Focus mode, intended for when a user has a specific goal, leading the interface to remove distractions and recommendation algorithms to facilitate a quick “in-and-out” experience, or an Explore mode, designed to broaden the user’s horizons by surfacing novel and diverse recommendations that encourage serendipitous discovery (Lukoff et al., 2018).

These proposals for improving IRs may partially address the objections of Krook and Blockx (2023) regarding the lack of active user involvement and the limited personalization of recommendations. The user could be interrupted, through different strategies, in their “blind” following of recommendations. But this raises a new problem: when does a friction cease to be positive? Even accepting that these cognitive “speed bumps” are positive in the pursuit of second-order desires, would the user accept such interruptions? Would they not lose incentives to use applications that operate with recommenders of this type, thereby depriving themselves of the potential benefits these could provide? In general, when frictions are introduced into a user–machine interface, the trade-off between the characteristic one wishes to enhance (for example, avoiding automation bias, facilitating the exploration of user preferences) and excessive paternalism—or, equally counterproductive, an interface design so unfriendly that it ends up not being used—must be carefully managed. Finally, we cannot ignore the elephant in the room: what incentive would the companies that develop or offer these recommenders have to include a less fluid interface with certain functionality that does not, a priori, seek a direct economic return?

In summary, in this section and the previous one, we have examined the problems we consider most important regarding the personal autonomy of RS users. These problems are more pronounced in what we have called Conjectural Recommenders. These systems make recommendations based on past behavior (both one’s own and that of similar people according to criteria not always known) and moreover assume that our desires are fully defined by that behavior—that is, by those choices. This type of design promotes the uncritical acceptance of proposed recommendations, which gives rise to three problems that we have described in detail. The first is that interaction with this type of system likely impedes the authentic development of preferences due to an excessive homogenization of recommendations. The second problem is related to the use of profiles created by the system with the aim of improving an objective that does not have to be aligned with the user’s typically increasing consumption or engagement. We have seen that this entails the impossibility of making sense of the proposed recommendations, which can have harmful effects on the development of personal identity. The third is the erosion of the deliberative capacities and willpower that we consider necessary for the exercise of our autonomy.

We have also introduced the concept of friction in the user interface and exemplified some positive frictions in what we have called Interrogative Recommenders. We have indicated that, although these frictions (whether IRs on their own or in hybrid models combining them with CRs) may have produced advances in the development of systems more respectful of user autonomy, they have not managed to fully mitigate the aforementioned effects and introduce new problems. For example, we have seen proposals that aim to disengage the user from a certain tendency toward first-order desires. These proposals, in a way, seek to prevent the user from doing what they initially want to do and what gives them pleasure. The first question to ask is whether we would not be being too paternalistic. If so, given our goal of promoting user autonomy, this would be a counterproductive irony that we should avoid through other, more appropriate designs. On the other hand, it must be acknowledged that, regardless of whether we are paternalistic or not, we are entering a territory where the lack of fluidity may become counterproductive. Can this be achieved without demanding so much effort from the user? Can we reach an adequate trade-off in which the RS offers an interface that is sufficiently fluid and attractive to users while at the same time helping them choose what they truly want to want? In the following section, we will present our proposal for a positive friction in the RS domain and attempt to respond to these challenges adequately.

## Socratic RS

In this section, we aim to present a sketch of recommender system design to help overcome the challenges identified in the previous section. A fundamental feature of our proposal is already considered in some recent projects: the use of dialogue between the system and the user as a key element in the process of generating recommendations. A “Conversational Recommender System” (CoRS) would combine the prediction of interests already found in CRs with dialogue through a virtual assistant that interacts with users and guides them through the different recommendations, allowing them to explore their preferences in a more reflective manner. This dialogue would be our proposed positive *first friction*: the objective is to disrupt the fluidity of using the CR and the uncritical selection of elements proposed by the recommender system, while simultaneously fostering in the user a conscious reflection on their own preferences.

In this recommender design, two techniques are combined. On the one hand, the recommendation is based on a knowledge base or collaborative filtering found in traditional recommender systems; on the other hand, using these initial recommendations as a first output, the capacity of new virtual assistants based on Large Language Models (LLM) to provide meaningful dialogue. The basic scheme typically involves the user initiating a conversation with a virtual assistant by requesting a recommendation for a specific type of item (e.g., films). The system will propose an initial recommendation based on traditional techniques, which will not be the final result, as in CRs, but rather the opposite: the starting point. From here, the assistant prompts the user to provide feedback on the recommendations, establishing a conversation in which the system captures the user’s preferences and thereby enriches the profile. It then proposes recommendations again based on this updated profile, incorporating input from the conversation. On the other hand, the fact that the user participates in the conversation reflects their interest in a more reflective use of recommendations rather than an impulsive acceptance of them. There are studies on the advantages of CoRS applications in specific domains such as book recommendations (Panda & Kaur, 2023; Lappalainen & Narayanan, 2023), healthcare recommendations (Nastasi et al., 2023; Thawkar et al., 2023), or hotel recommendations (Remountakis et al., 2023).

Because they can generate an *apparently* meaningful and enriching conversation for the user, these assistant-recommenders can engage in dialogues that are very similar to those maintained by humans. They can generate responses that adapt to the intentions and nuances of users’ requests, thus facilitating real-time recommendations that, because they result from dynamic and more engaging interactions, are also more personalized and convincing (Vaswani et al., 2017; Brown et al., 2020; Rima et al., 2023; Omara et al., 2023; Sohail et al., 2023; Kalirai & Kuzminykh, 2024). This dynamic interaction—requesting, when necessary, that issues be clarified and that the nuances of the conversation be reflected—leads to a highly immersive user experience in which the recommendations are seamlessly integrated into the dialogues, improving user satisfaction and engagement (Vaswani et al., 2017; Brown et al., 2020; Kalirai & Kuzminykh, 2024; Al-Hasan et al., 2024). The user would end up accepting this type of system more, not only because of the interactive process itself but also because of the results, insofar as they perceive the recommendations as closer to their real-world experiences.

This is because, to achieve its objective, the system is provided with a new input, the conversation, which is integrated with other existing data, such as social media interactions, Internet of Things devices, location-based services, and more, considerably enriching the user profile managed by the system (Vaswani et al., 2017).

In addition, this design enables users to explore their higher-level preferences, as it allows recommendation systems to treat user preferences as dynamic, stratified, and subject to negotiation that reconciles conflicting desires or goals, thereby reflecting the social aspects that typically intervene in the human recommendation process. This is especially useful in scenarios where the user does not know exactly what they want, or where their preferences are unstable or complex (Kalirai & Kuzminykh, 2024). But it is not only useful for that reason. Offering with the dialogue a narrative that the user could accept as a reason for their preference would be an essential factor for improving recommendations. In real life, several factors influence someone’s decision. Thus, for example, a user might want to acquire a product out of curiosity or due to the influence of others. Or it might be a desire resulting from a firm, well-considered decision. Therefore, different people may acquire the same product for different reasons. One should not, as traditional systems do, treat users equally who, while manifesting the same external desire for something, have very different reasons for desiring it. CoRSs could represent a significant advance by noting this and seeking to provide users recommedations with which one can best identify rationally and in terms of identity.

The possibilities opened by these CoRSs may yield significant advances in efficiency, recommendation updating, and personalization, and may foster user reflection on their preferences. However, although this dialogue is necessary for safeguarding and enhancing user autonomy, it does not seem sufficient in itself. This is because, from our perspective, despite constituting positive friction in the system’s design, this dialogue does not yet ensure that preferences are the result of reflection, nor does it guarantee personal development through pluralism. The recommendation may respond to a dialogue around what the user says they want at that moment but is it what they want to want? The question we posed regarding traditional systems remains relevant. Just as it continues to make sense to maintain that these recommenders do not guarantee that the degradation of reflective capacities would be avoided. Certainly, dialogue can represent an advance over traditional systems by requiring the user to take a more personalized stance by responding to more specific questions, but that alone does not require the user to resort to reflection to answer them. The user’s responses could follow a fixed, highly limited pattern restricted to strong first-order desires.

To break out of this dynamic, we propose a *second friction* (in addition to that involved in the dialogue itself): that the chatbot be more inquisitive and formulate its questions according to certain patterns oriented toward seeking greater reflection on what is preferred. In other words, the chatbot should attempt to facilitate for the user a more appropiate departure from the inertia of immediate and automatic preferences.

We believe that advancing in this direction of bringing CoRSs closer to autonomy can be supported by the ideas proposed by F. Lara and J. Deckers for the design of a virtual assistant that, inspired by the Socratic method, would morally enhance human beings (Lara & Deckers, 2020; Lara, 2021).^2^ The distinction between an *advisor* model of AI-assisted enhancenment, which supplies the user with substantive recommendations, and an *interlocutor* or *Socratic* model, which instead elicits the user’s own reasoning, was itself anticipated by Klincewicz (2016), who raised the autonomy costs of the former in the context of AI-based moral enhancement. Lara and Deckers (2020) build on this distinction in designing the model we are going to adopt here in extending the interlocutor model to the domain of recommendation. This assistant, known as SocrAI, in contrast to other models of moral enhancement, would adopt a scheme that, rather than advising the user, helps them advance their learning of moral deliberation through interaction with the assistant. The chatbot would serve only as an interlocutor, not providing moral solutions to the user; rather, by questioning the user’s statements, it would, like the Socratic midwife, help the user arrive at possible answers to their moral questions on their own. From this basic scheme, the fine-tuned training of SocrAI with specialized sources and its conversational interface would be designed to help the user’s arguments achieve empirical rigor, conceptual clarity, argumentative logic, and practical feasibility, enabling the assistant’s constant questions to serve as a stimulus for the user to reflect on their moral positions and, through this, to enter a process of learning and better use of their moral capacities. The choice of this SocrAI over its advisory rivals takes a stance within the autonomy trade-off that has structured the debate on artificial moral enhancement at least since Klincewicz (2016). An advisory system, i.e., an artificial moral reasoning engine presenting users with arguments grounded in normative theories, may well be more effective at changing behavior, precisely because it can exploit the trust, sometimes undue, that users place in automated technologies. But in the recommender context, that effectiveness leverages on the very dynamics of uncritical acceptance that we criticized in CRs in Section “Autonomy and Conjectural Recommenders”.

We believe that this virtual assistant model could inspire a recommender that is more efficient in terms of autonomy. We will call it the “Socratic CoRS.” Its essential objective would be, following the approach of SocrAI, to focus the discussion on one’s own second-order desires, rather than on the particular recommendations in which they manifest. It is through this dialogue about second-order desires, or metapreferences, linked to individual recommendations, or first-order desires, that we come closer to what the user wants to want, and in turn foster their autonomy.

To this end, and drawing inspiration from the aforementioned SocrAI, it would be important for the system to be designed to maximise user autonomy in the formation of their preferences. This requires, above all, that the system should under no circumstances provide users with substantive proposals as to what would be a good preference. Its functional criteria must be strictly formal, aiming for the improvement of preferences to be, to some degree, the result of a process in which the user has reflected on their desires, and has done so according to criteria such as personal coherence and integrity, empirical and consequential knowledge of their preferences, and choosing from cognitive pluralism. In the terms developed below, these criteria are formal in that they leave untouched who is doing the deliberating, while still shaping the conditions under which that deliberation counts as reliable.

It should be acknowledged that describing these criteria as “formal” does not amount to claiming that they are normatively inert. Any account of what counts as good deliberation, however minimal, carries some commitment about the architecture of rational agency; this is a familiar difficulty for every procedural theory of autonomy, not a peculiarity of our proposal (Christman, 2020). The relevant contrast is not between a neutral and a value-laden design, but between a design that commits only to the *conditions* under which a preference can be considered the agent’s own—that it be examined, that it be responsive to evidence, that it survive confrontation with alternatives—and one that commits to *which* preferences the agent should end up holding. The Socratic CoRS is substantive in the first, thin sense and remains agnostic in the second, thicker sense that is relevant to the charge of paternalism.

This distinction also addresses a natural objection to cognitive pluralism as a design criterion: users are routinely exposed to opposing viewpoints in ordinary digital life, apparently with little corrective effect, a fact well documented by the literature on selective exposure and confirmation bias (Nickerson, 1998; Taber & Lodge, 2006; Bakshy et al., 2015). Worden (2019) clarifies why mere exposure falls short: distinguishing, after Nguyen (2018), between epistemic bubbles—where alternative views are unwittingly absent—and echo chambers—where they are actively delegitimized even when present, she argues that inclusivity (contact with diverse viewpoints) must be paired with a distinct virtue of self-control, understood as the deliberate monitoring of one’s own cognitive biases while engaging with that content, for exposure to yield any corrective benefit. Incidental exposure to disagreement, without this further step, is compatible with an echo chamber in Nguyen’s sense: the alternative view is present but never genuinely engaged. It is worth noting that not every virtue-theoretic account of online engagement converges on a general preference for confrontation over avoidance: Elder (2014) defends disengagement—unfollowing or blocking—as legitimate when the relationship in question is a loose social tie. Yet she draws a sharp line at closer, more significant relationships, where avoiding a disagreement “at the cost of honesty and openness” can be, in her own words, a form of cowardice. The tension the Socratic CoRS surfaces is not a disagreement with a third party whose views the user might reasonably set aside, but an internal tension between the user’s own stated preferences—the case structurally closest, on Elder’s own distinction, to the one where avoidance is least defensible. The system does not merely surface a plurality of contents alongside the user’s own; it actively identifies this internal tension and requires the user to resolve or rank it, as the dialogue that follows illustrates, between preferences 1 and 3—the functional analogue, within a recommender system, of Worden’s self-control.

A similar clarification is needed regarding coherence as a deliberative virtue. We do not intend coherence in the strong coherentist sense long criticized in epistemology for allowing a belief system—or, here, a preference system—to remain internally consistent while disconnected from experience (Sosa, 1980). This is why coherence is paired, in our design, with empirical and consequential awareness, which supplies the corrective role that pure coherentism lacks on its own.

The point can be put more precisely using the tripartite analysis of competences that Sosa develops in virtue epistemology, and that Klincewicz (2025) applies to preparatory moral technologies: a competence is exercised reliably when the right *seat*—the subject in whom the competence resides—acts in the right *situation*, drawing on the right internal *structure*. What distinguishes preparatory technologies, such as Stoic robotic nudges or the Socratic assistant, from substitutive ones is that they leave the seat of moral or deliberative agency untouched: the user, not the system, remains the one who judges. What they legitimately intervene on is situation and structure. On this mapping, cognitive pluralism operates on the *situation*—ensuring the user deliberates in confrontation with relevant alternatives rather than in an epistemically impoverished environment—while coherence operates on the *structure*—ensuring the user’s own reasoning meets a minimal condition of internal consistency once that confrontation has taken place. Neither criterion specifies which preference the user’s seat should ultimately endorse; both specify conditions under which the user’s own exercise of that capacity counts as reliable rather than arbitrary. This is precisely the sense in which the Socratic CoRS’s criteria are formal: they constrain the reliability of the process without constraining its output.

This choice of a reliabilist rendering of coherence, rather than a responsibilist one, is deliberate. On a responsibilist account of intellectual virtue, possessing a virtue requires both a competence and a motivation to exercise it well (Zagzebski, 1996; Baehr, 2016; Kotsonis, 2023), and, following Baehr (2013), every intellectual virtue can further be seen as pursuing an ultimate goal common to all virtues (reliable cognitive contact with one’s own values) through an immediate goal distinctive of each virtue—coherence’s immediate goal being internal consistency among one’s reasons, cognitive pluralism’s being confrontation with relevant alternatives (see Kotsonis, 2023, for this distinction applied to an overarching intellectual virtue). A system such as the Socratic CoRS has no legitimate basis for supplying or shaping the user’s *motivation* to deliberate well; doing so would reintroduce exactly the kind of substantive commitment the design is meant to avoid. What it can legitimately support is the *competence* component alone—the reliable exercise of a motivation to deliberate that the user is assumed already to have. This is a further sense in which the system’s criteria remain formal: they scaffold the exercise of a virtue whose motivational core stays entirely the user’s own.

This second friction that we propose to improve the CoRSs would start from the previously described design—that is, one in which the system provides a list of recommendations based on traditional recommendation techniques, upon which a dialogue would begin. However, in this dialogue, our assistant would focus on the preferences that those recommendations reflect. To this end, we propose to fine-tune a generic model on user–system interactions, as exemplified below, and leverage the generalization capabilities of LLMs to generate meaningful discussions in other domains^3^.

**User:**I’m looking for a movie to watch tonight.

**System (Recommender):** Before suggesting what to watch, I’m going to propose the preferences that seem to be reflected in your previous choices. Tell me where I get it right or where I am wrong.

1. “Let philosophically rich films take precedence over easy entertainment.”
2. “Prioritize human–technology inquiry when choosing science fiction.”
3. “Prefer influential works over trendy releases.”
4. “Accept ambiguity and moral complexity rather than a neat ending.”
5. “Choose speculations that illuminate present social life.”

**User:**That’s pretty close, but I’m not sure I identify with number 2. Could you elaborate more on what we’re referring to?

**System:**Yes, I mean that when several sci-fi options compete, you prefer to watch works that explore how technology reconfigures the human (identity, agency, intimacy, memory, power, work), over alternatives focused on action or spectacle.

**User:**No, I also prefer visually appealing films with plenty of action.

**System:**All right, in that case, I would add a new preference—entertainment and visual effects:

1. “Let philosophically rich films take precedence over easy entertainment.”
2. “Prioritize human–technology inquiry when choosing science fiction.”
3. “Entertainment and visual effects.”
4. “Prefer influential works over trendy releases.”
5. “Accept ambiguity and moral complexity rather than a neat ending.”
6. “Choose speculations that illuminate present social life.”

It appears there is a tension among those preferences, particularly between 1 and 3. Unless you provide a ranking of importance, I will assign the same weight to all.

**User:**The first is the most important. The rest have similar importance.

**System:**All right, I will now proceed to present my recommendations based on that information.

It is now time to consider the extent to which this Socratic CoRS proposal could better address the autonomy challenges posed to traditional models, as set out above.

Firstly, it should be made clear that the objective of conversational recommenders, both CoRS and Socratic CoRS, is not to identify the user’s true second-order desires—we do not believe that LLMs are equipped for this—but rather to foster conscious user reflection on them. This alone would substantially improve the problems we analyzed in CRs, whose main characteristic is interface fluidity and the consequent pursuit of uncritical acceptance of proposed recommendations. We saw that this led to excessive homogenization of recommendations and made it difficult for the user to make sense of the proposals, which we consider necessary for the adequate development of personal identity. With CoRSs, this simply does not occur, since the presentation of recommendations is the first step upon which to develop the conversation. This would also mitigate, though not fully resolve, the absence of social interactions and their role as a self-correction mechanism.^4^ The Socratic version, moreover, focuses directly on the second-order desires related to those recommendations and facilitates the user’s reflective endorsement of their own metapreferences, thereby mitigating problems in developing an authentic identity, or at least one not surreptitiously influenced.

Regarding the problems introduced by IRs, we saw that these proposals can fall into unacceptable paternalism or, equally counterproductive, disincentivize the use of these systems. The CoRS could be accused of paternalism if the assistant’s participation were aimed at directing the user toward certain preferences considered better for them. However, in the proposal presented for the Socratic CoRS, if the user is directed anywhere, it can only be toward fulfilling the formal requirement that their preferences be truly their own and free. This is because what is fostered is a discussion that is as unbiased as possible about one’s own preferences, seeking to center the discussion on the user’s second-order desires—that is, on jointly searching for what the user wants to want rather than what they want. And all of this, while adhering to criteria that are also formal, such as personal coherence and integrity, empirical and consequential knowledge of one’s preferences, and choosing from cognitive pluralism, which can hardly be considered a condemnable paternalism.

Perhaps the problem of disincentivizing use is among the most complex issues to address in the Socratic CoRS proposal, particularly given the widespread tendency to provide users with highly fluid interfaces. Would anyone accept being periodically subjected to a Socratic-style interrogation to clarify their preferences? This is particularly true if we consider both the uncomfortable process of having to stop and think and the fact that the outcome will probably entail leaving the comfort zone in which the user is carried along by their tendency to satisfy basic first-order desires, which are usually more pleasurable in the short term. It is conceivable that the user might ultimately prefer applications with less demanding and more fluid recommenders.

Nevertheless, there are factors to consider that may partially counter this objection. First, the system’s reliability, given its educational character and its relevance to alerting to potential risks to autonomy, which are not easily perceived in the use of other systems. Some specialists have maintained that an effective strategy for mitigating risks associated with the use of algorithmic systems is to provide education and training on how these systems work, specifically in the case at hand, on the reasons behind algorithmic recommendations (Danaher, 2018; Höller et al., 2023; Ienca, 2023). Along the same lines, Bartmann (2023, p. 130) insists that only if we understand why certain recommendations are presented to us and not others will it be possible to integrate them into decision-making in a way that preserves autonomy. Thus, the user may perceive the process as similar to a human recommendation, with an explanation of the reasons and possible interaction regarding the real interests of the person being recommended to. Lu (2024) has responded to this strategy by correctly pointing out the difficulty of implementing it, since AI lacks the capacity for rational reasoning and can only generate results based on probabilities.

Although from our point of view the dialogue we propose would not alleviate this difficulty, given that the system would remain a “stochastic parrot” (Bender et al., 2021), the functionality for which we would train it would be to foster active reflection in the user. It is worth noting that our stand on the epistemic status of the system, or whether Bender et al. (2021)’s deflationary view is ultimately correct, is not important here. What matters for our argument is that nothing in our proposal requires the system to possess or understand reasons. The epistemic work, on our account, is done entirely by the user; whatever effect the system has proceeds through the user’s own generation and examination of reasons. Readers who reject the assumption may substitute a richer account of LLM capacities without loss to the argument; readers who accept it need only grant that statistically generated questions can prompt genuine reflection. In this way, what matters is not the final recommendation or the reasons the system may have had for proposing it, but the user’s reflection on their own reasons for preferring something, and the user’s confirmation that the recommendations align with them.

We believe that this functionality would be attractive to a large number of people and, in any case, in order to enhance its appeal, we would consider the attenuation of its implementation to a certain degree and even seeing it as an optional feature that could be deactivated if the user so requires.

Second, there is the persuasive power of reason. Although it is true that, as Hume argued, it is desires and emotions that motivate us—as opposed to the inert character of an argument by itself—it is also true that desires can twice motivate us when accompanied by reasons (they cease to be blind in Humean terms) (Lara, 2021, p. 20). This is what would occur with the preferences resulting from the dialogue between the system and the user.

Finally, those more motivating preferences—by virtue of the rational support that accompanies them—could even further increase their motivational power if their holders are aware that the reason supporting these preferences does not come from an external source, but from the users themselves as agents, and in particular from an arduous and participatory process based on the dialogue between them and the assistant (Lara, 2021, p. 21). Moreover, the conversational recommendation process itself, being perceived as an essentially interactive initiative between users and the system, can generate greater trust (at least among users with domain knowledge and greater conscientiousness) (Cai et al., 2022).

## Conclusion

This article has examined the relationship between recommender systems and human autonomy, arguing that the dominant design paradigms in current RSs pose significant threats to the authentic formation and development of user preferences. We have shown that these threats operate at multiple levels: through the narrowing of the preference landscape, the erosion of deliberative capacities, and the impediment to personal identity development.

This is particularly true of the systems we call Conjectural Recommenders (CRs), which rely on implicit behavioral data to generate predictions, systematically undermining autonomy by conflating users’ observed choices with their genuine preferences. By ignoring the gap between first-order desires and the deeper second-order desires that constitute authentic autonomy in the Frankfurtian and Dworkinian sense, CRs trap users in homogeneous digital environments that reinforce past behavior, inhibit identity development, and progressively degrade the very deliberative capacities required for human autonomy.

We have introduced the concept of positive friction, which we have applied to what we have called Interrogative Recommenders (IRs)—systems that incorporate explicit user feedback. While these represent an advance by prompting users to articulate their evaluations, we argued that they remain insufficient for accessing second-order desires. We have also introduced a trade-off in the design of positive frictions in this domain: the more friction we introduce, the greater the risk of either paternalism or user disengagement. This balance constitutes a central design challenge for any RS that aspires to protect autonomy.

Our principal contribution has been to propose the Socratic CoRS as a two-layered friction design that addresses the limitations identified in both CRs and IRs. The first friction, the conversational element, disrupts uncritical acceptance of recommendations by turning them into starting points for dialogue. The second friction, the Socratic orientation, goes further by directing that dialogue toward the user’s metapreferences, employing only formal criteria (coherence, empirical knowledge, cognitive pluralism) rather than substantive conceptions of the good. The goal is not for the system to discover the user’s “true” second-order desires—an epistemic task we do not attribute to LLMs—but to foster the user’s own reflective engagement with their metapreferences. This design avoids the charge of paternalism precisely because it does not prescribe what users should prefer, but rather fosters the conditions under which preferences can be authentically formed. Regarding the disincentive problem, we argue that the persuasive power of reason-backed preferences, the sense of ownership over deliberatively formed choices, and the trust generated by a process that mirrors human recommendation practices offer plausible grounds for user engagement.

Several limitations of this proposal should be acknowledged. First, as we have conceded, the system does not resolve the problem identified by Lu (2024) regarding the irreplaceable role of genuine social interactions as a corrective mechanism for autonomous decision-making. Second, the tension between autonomy-enhancing friction and user acceptance remains unresolved. Market incentives for companies to adopt such designs are uncertain, especially in environments driven by attention and engagement metrics. Finally, the reliance on LLMs—which, as “stochastic parrots,” lack genuine rational capacities—means that the quality of the Socratic dialogue will always depend on the sophistication of the fine-tuning and the user’s own willingness to engage.

Despite these open challenges, we contend that the transition toward an autonomy-sensitive framework for recommender systems is both a normative necessity and a practical imperative. As algorithmic mediation increasingly governs the informational landscape of our daily lives, the central question is no longer whether these architectures influence human agency, but rather how they ought to be designed to safeguard it. The Socratic Conversational Recommender represents a substantial advancement in embedding deliberative agency within the very logic of recommendation. By transcending traditional models that merely extrapolate future behavior from past data, the Socratic approach ensures that technology functions not as a catalyst for impulsive consumption, but as a medium for self-reflection. Ultimately, such systems empower users to exercise true digital autonomy, enabling them to choose not merely on the basis of immediate, first-order preferences, but through an alignment with their metapreferences, effectively bridging the gap between what we happen to desire and what we truly want to want.

## Data Availability

No datasets were generated or analyzed during the current study. The analysis is based entirely on existing literature cited within the reference section.

## Footnotes

1 Here we are referring to a minimal conception without disparaging other, more sophisticated approaches to autonomy—particularly those that treat autonomy as a relational capacity (Mackenzie, 2008) or other hybrid approaches that have been proposed in the specific case of recommender systems (del Valle & Lara, 2023).

2 Lu (2024), in his defense of the position that algorithmic decision-making systems should be designed so as to always operate from the idea of “human-in-the-loop,” concludes his article by very briefly pointing out—since, as he says, it would go beyond the scope of his article—the relevance that the Socratic AI model of Lara (2021) would have for this purpose.

3 Generalization refers to the capacity of Large Language Models to perform competently across a wide range of domains and tasks without being specifically trained for each of them, unlike traditional narrow AI systems designed for a single purpose.

4 We do not believe that any of the options presented resolves the criticism of W. Lu (2024) regarding the need for social interactions as a self-corrective mechanism, nor do we of course claim that the conversation we propose here is a replacement for them.
